# Reproductive Performance of Zi-Goose Promoted by Red Color Illumination

**DOI:** 10.3389/fvets.2022.879478

**Published:** 2022-11-24

**Authors:** Manyu Li, Chen Liang, Xiuhua Zhao, Guojun Liu, Yuanliang Zhang, Shan Yue, Zhiqiang Zhang

**Affiliations:** ^1^Institute of Animal Husbandry, Heilongjiang Academy of Agricultural Sciences, Harbin, China; ^2^College of Pharmacy, Henan University of Traditional Chinese Medicine, Zhengzhou, China

**Keywords:** red light, reproductive, transcriptome, Zi-goose, hypothalamus

## Abstract

The color of light affects the reproductive performance of poultry, but it is not clear what efficient illumination strategy could be adopted to improve the reproductive performance of Zi-goose. Red light can increase the average weekly egg production rate, egg production, and qualified production. It can increase the serum GnRH level and decrease the serum PRL, MT, and T4 levels. In our study, red light for 12 h increased the average weekly laying rate, average qualified egg production, and hatching rate of Zi-goose eggs, and increased the serum levels of FSH, LH, P4, E2, MT, T3, and T4. Blue light at 14 h improved the average weekly egg production rate, average egg production, and average qualified egg production, and reduce serum PRL and MT levels to ensure the improvement of reproductive performance of goose. A total of 705,714 overlapping group sequences, 471,145 transcript sequences, and 268,609 single gene sequences were obtained from 18 sequencing samples, with a total length of 323.04, 668.53, and 247.88 M, respectively. About 176,416 unigenes were annotated successfully in six databases, accounting for 65.68% of the total unigenes obtained. 2,106, 2,142, and 8,892 unigenes were identified in the hypothalamus, pituitary gland, and ovary of the birds respectively, with different expressions of light regulation. The hypothalamus, ovary, and pituitary were involved in 279, 327, and 275 KEGG (Kyoto Encyclopedia of Genes and Genomes) metabolic pathways in response to light, respectively. Through further significance analysis and differential discovery rate control, a total of five metabolic pathways were obtained which were closely related to the reproductive hormones of goose. Ten candidate genes related to the reproductive performance of goslings were selected according to the identification results of differentially expressed genes of goslings under red light and white light conditions and the genes involved in metabolic pathways significantly related to the reproductive hormones of goslings. The expression levels of *GnRh-1* in the hypothalamus, *GnRH-R, FSH* *β* and *LH* *β* in the pituitary gland, and *FSH-R* and *LH-R* candidate genes in the ovary were higher under the 12 h red light treatment than white light. However, the expression levels of *VIP, PRL*, and *PRL-R* candidate genes in the hypothalamus, pituitary and ovary were lower under 12 h red light than under 12 h white light.

## Introduction

Lighting is essential in avian species, such as manipulating reproductive performance and sexual maturity (1). Currently, artificial light supplements have become a major measure to improve the reproductive performance of poultry in modern domestic fowl production (2). It is known that different colors of light have different wavelengths and long wavelength lights are perceived more easily by birds than the short wavelength lights, though the light intensities remain the same (3). Generally, red light of long wavelength has a favorable effect on egg production and the onset of laying, while blue or green light of short wavelength had a negative effect on egg production but had an accelerating effect on growth (4, 5). To our knowledge, a majority of research on this aspect was focused on hens, ducks, and turkeys along with rabbits, sheep, camels, and so on (6). However, scarce information is available on the impact of light color on the reproductive performance of goose. Moreover, it is also not clear how the endocrine hormones and their gene affect the reproductive performance of goose exposed to different color light sources. As for changes in the transcriptomic profile of goose gonads in response to different color lighting, we can hardly find any literature.

As a result of photostimulation, hypothalamic photoreceptors in avian species initiate a photo-sexual response that controls their reproductive performance through the hypothalamic-pituitary-ovarian gonadal axis. Longer wavelengths of light (such as red) penetrate through the feathers, skull, and cranial tissues to the hypothalamus more efficiently than lights of shorter wavelengths (such as blue), and have a great influence on the reproductive performance of birds (7, 8). Building on the findings of Baxter et al. (9) that the reproductive axis in chickens was activated by red light and this phenomenon occurred independent of the involvement of the hen's retina, we hypothesized that red light will greatly affect the reproductive performance of goose by regulating the secretion of their reproductive hormones and the expression of reproduction-related genes (8, 9).

This research aims at assessing the efficacy of colored lighting that improves the reproductive performance of goose, uncovering the responses of the reproductive hormones and expression of reproduction-related genes to different color lighting conditions. Our study also looks at differentiating the changes in the transcriptomic profile of the hypothalamic gonad of goose.

## Materials and Methods

### Ethics Statement

This study was conducted in strict accordance with the guidelines of the Animal Ethical Welfare Committee. The research protocol was reviewed and approved by the Animal Care and Use Committee of Northeast Agricultural University.

### Animals and Experiment Design

A total of 450 Zi-goose (1 year old) were randomly distributed into three groups with three replicates per group and 10 males and 40 females per replicate. The grouping was based on the color of the light: blue LED group, red LED group, and white LED group, which served as the control. The birds were housed inside the chambers that provided a stocking density of 5.0 birds/m^2^ and exposed to different color LED illumination for 12 h lighting per day from 1 March to 30 June 2018. Routine feeding and management were adopted during the whole experiment. The diet compositions of the experiment are shown in Table 1.

**Table 1:** The diet composition of experiment.

**Diet composition**	**Contents (%)**	**Nutrient components^B^**	**Content**
Corn	3.00	Metabolizable energy (MJ/kg)	10.21
Maize starch	39.00	Crude protein (CP) (g/kg)	15.60
Corn germ meal	18.00	Total Calcium (g/kg)	1.72
Soybean oil	0.50	Total phosphorus (g/kg)	0.85
Rice bran meal	16.00	Total lysine (g/kg)	0.70
Soybean meal	12.00	Total methionine (g/kg)	0.50
Chili meal	3.00	Crude fiber (g/kg)	4.81
DDG distillers dried grains	0.50		
Salt	0.35		
Dicalcium phosphate	1.10		
Limestone	3.50		
Methionine	0.35		
Choline	0.30		
Chinese herbal medicine	1.00		
Premix^a^	2.00		

a Premix can provide 10 mg of copper, 90 mg of zinc, 0.1 mg of selenium, 15 mg of manganese, 60 mg of iron, 0.1 mg of iodine, 4000 IU of vitamin A, 600 IU of vitamin D3, 0.1 mg of vitamin K, 0.6 mg of vitamin B2, 0.4 mg of pantothenic acid, 0.6 mg of nicotinic acid, 2.6 mg of vitamin C, 0.04 mg of folic acid, 60.06 mg of vitamin B and 0.06 mg of vitamin B1 for 1 kg diet;

b Values of nutrient components are gained by calculation.

### Determination of Reproductive Performance Indexes

The eggs were collected every day from each group and sorted into qualified and unqualified eggs, which were then used for calculating weekly averages of egg-laying, qualified egg-laying, and egg-laying rates. In May, qualified eggs within a week of their incubation were tested to determine the weekly average fertilization and hatching rates.

Weekly egg-laying numbers = Egg-laying numbers in seven consecutive days/seven.

Weekly qualified egg-laying numbers = Qualified egg-laying numbers in seven consecutive days/seven.

Weekly average egg-laying rate (%) = Egg-laying numbers in a week/average female goose numbers × 100%.

Average fertilization rate (%) = Number of fertilized eggs/hatching eggs in a week × 100%.

Average hatching rate (%) = Number of hatchlings/Number of fertilized eggs in a week × 100%.

Three birds in each treatment were slaughtered randomly and the hypothalamus, ovary, and pituitary gland tissues were taken and frozen immediately in liquid nitrogen and stored at −80°C for quantitative real-time PCR analysis of the reproduction-related genes. Simultaneously, the hypothalamus tissues treated with red LED and white LED were also used for transcriptome sequencing analysis.

### Determination of Reproductive Hormone Levels in Serum

The blood samples from the pterygoid vein of four female birds for each replicate were collected to measure the reproductive hormone levels in serum on 20 March and 28 May 2018 respectively. After coagulation, the blood samples were centrifuged at 3,000 rpm for 10 min, and the serums were collected respectively. The levels of Gonadotropin-Releasing Hormone (GnRH), Prolactin (PRL), Follicle-Stimulating Hormone (FSH), Luteinizing Hormone (LH), Estradiol (E2), Progesterone (P4), Melatonin (MT), Triiodothyronine (T3), and Thyroxine (T4) in serum were determined by ELISA assay according to the quantitative diagnostic kit of estradiol produced by North Institute of Biotechnology (Beijing). The assay sensitivity were 0.1 mIU/ml, 1.0 ng/ml, 1.0 pg/ml, 0.1 ng/ml, 1.0 ng/ml, 1.0 mIU/L, 0.1 nmol/L, and 1.0 nmol/L, respectively. The coefficient of variation among individuals and replicates was <15%. The serum samples were continuously diluted to obtain the inhibition curve parallel to the standard curve. The R-value of the measurement curve and the standard curve was more than 0.99.

### The Expression Levels of Reproduction-Related Genes Detected by RT-PCR

In this study, the expression levels of reproduction-related genes in the hypothalamus, pituitary, and ovary gland tissues, such as GnRH Receptor (GnRH-R), GnRH Inhibitor (GnRH-I), Gonadotropin-Inhibiting Hormone Receptor (GnIH-R), VIP, FSH beta, LH beta, FSH Receptor (FSH-R), LH Receptor (LH-R), PRL, and PRL Receptor (PRL-*R)*, were detected by RT-qPCR using *β-actin* as an internal standard gene. The RT-qPCR was conducted in 20 μl reactions that each contained 10 μl 2× SYBR Premix Ex Taq, 1 μl cDNA, 0.5 μl upstream primer (10 μM), 0.5 μl downstream primer (10 μM), and 8 μl sterile distilled H_2_O. An ABI PRISM-7500 sequence detection system was used to detect the amplified products. Gene expression levels were expressed using the 2^−ΔΔCT^ method (10) and were normalized to the expression levels of *β-actin*, an internal housekeeping gene.

### Transcriptome Sequencing Analysis

#### RNA Extraction and Detection

Total RNA was extracted by Trizol one-step extraction method, and the samples were treated with DNase I to remove DNA contamination. The integrity of RNA was detected by 1% agarose gel electrophoresis. RNA purity and concentration were detected by NanoDrop 1000.

#### Construction and Library Inspection of CDNA Library

The mRNA with polyA structure in total RNA was enriched by Oligo (dT) magnetic beads, and the RNA was interrupted to about 300 bp in length by ion interruption. The fragment with a length of 300 bp was selected because the length of the joint was fixed. Using RNA as a template, the first cDNA strand was synthesized with 6-base random primers and reverse transcriptase, and the second cDNA strand was synthesized with the first cDNA strand as a template.

After the library was constructed, the library fragments were enriched using PCR amplification. Then, the total concentration and effective concentration of the library were detected through Agilent 2100 Bioanalyzer. According to the effective concentration of the library and the amount of data required by the library, the library containing different index sequences was mixed in proportion. The mixed library was uniformly diluted to 2 nM, and a single strand library was formed by alkali denaturation.

#### Quality Control of Sequencing Data

The short sequences obtained by sequencing are generally raw data. Because redundant or low-quality data, such as low base recognition, specific sequence interference, PCR non-specific amplification, and sequencing joint contamination often occur in RNA extraction, during library preparation and sequencing, the reads with these quality problems get filtered out through quality assessment and control to ensure the normal conduct of subsequent bioinformatics analysis.

#### Quality Control Data Splicing

Clean Reads were spliced with Trinity software to obtain transcripts and then analyzed. Trinity is a *de novo* assembly software for transcriptome splicing, which splices high-quality sequences based on DBG (De Bruijn Graph) splicing principle. The software is composed of three independent software modules, and the workflow of these three modules is as follows:

1) Inchworm: constructs a short sequence library of k-mer length by using high-quality sequences, and extends the short sequence by k-mer-1 length. It then overlaps between short sequences to obtain a preliminary contig sequence.
2) Chrysalis: clusters them through overlap between contig sequences, and then constructs Bruijn graph for each class.
3) Butterfly: processes these Bruijn diagrams, finding pathways according to Reads and paired Reads in the diagram, and obtains transcripts.
4) After stitching, transcript sequence files in FASTA format can be obtained. The longest transcript under each gene is extracted as the representative sequence of the gene, called unigene.

#### Functional Annotation and Classification of Unigene

In order to clarify the biological functions of unigene, Blast analysis was performed on the unigene obtained from different databases, following which gene function annotation was performed.

#### Screening and Analysis of Differential Expression of Unigene

Of the gene expressed by DESeq variance analysis, screening of differentially expressed gene conditions were: multiple expression differences|log_2_FoldChange|>1, significance *P* < 0.05. In order to explore the differentially expressed genes between unknown biological contact, we used R language Pheatmap packages to compare the difference between the set of genes and sets and two-way clustering analysis samples, according to the same gene expression level in different samples and different gene expression patterns in the same sample clustering, using the method of Euclidean distance. Complete Linkage was used for clustering.

#### Gene Ontology Enrichment Analysis of Differentially Expressed Genes

TopGO was used for Gene Ontology (GO) enrichment analysis. During the analysis, the gene list and gene number of each term were calculated using the differential genes annotated by GO term. The *P*-value was calculated by the hypergeometric distribution method to find out the GO terms with significant enrichment of differential genes compared with the whole genome background. Thus the main biological functions performed by the differential genes were determined.

#### KEGG Enrichment Analysis and KEGG Orthology (KO) Analysis of Differentially Expressed Genes

According to KEGG enrichment results, the enrichment degree was measured by Rich Factor, FDR value, and the number of genes enriched into this pathway. Rich factor refers to the ratio of the number of differential genes enriched and annotated in this pathway. The greater the Rich factor, the greater the degree of enrichment. KO and Pathway annotations mainly use the KEGG KAAS (KEGG Automatic Annotation Server) (http://www.genome.jp/tools/kaas/). Related species were selected for gene sets, and Bi-directional Best Hit (BBH) was used for gene KO identification.

#### RNA-Seq Data Validation

Candidate genes were screened from reproductive hormone-related metabolic pathways in goose, and *β-actin* was used as an internal reference gene to verify transcriptome data by RT-qPCR. Primer Premier 5.0 software was used to design target gene primers, and practical target gene primers were screened by Real-time PCR (Table 2).

**Table 2:** Primers corresponding to the purpose genes used in the real-time quantitative PCR assay.

**Gene name**	**Primer sequence(5^′^-3^′^)**	**Annealing** **temperature** **(°C)**	**PCR** **product (bp)**
*β-actin*	F: CCA AAG CCA ACA GAG AGA AG	60	159
	R: TCA CCA GAG TCC ATC ACA ATA C		
*GnRH-I*	F: CTG GGA CTT CAC AGA CCT AAC	60	232
	R: GGA CTT CCA ACC ATC ACT G		
*VIP*	F: GGG CTA AAC TTG CTG TGA	58	204
	R: GAA AGC GGC TGT AGT TGT C		
*GnRH-R*	F: GGT CAT CGT CTC CTC CTT CAT C	60	232
	R: CCA GGC AGG CAT TGA AGA G		
*GnIH-R*	F: GCC CTC ATC GTC GTC ATG TA	58	186
	R: AGA CAC CTT CCT CCC CTC AG		
*PRL*	F: CAG CAG ATT CAC CAT GAA GAC	56	218
	R: CAA TGT CGC CAG AAT GAA C		
*FSH beta*	F: AGC AGT GGA AAG AGA AGA ATG TG	60	209
	R: ACC GTT CAG ACT GTC AAT GTA TCTA		
*LH beta*	F: CCC CAA TGT ATG GCT GTG	58	90
	R: CAA AGG GCT GCG ATA CAC		
*LH-R*	F: TCT GAA GGA CAA CAG AAA CCT C	58	179
	R: CCA GTG CCG TTG AAG AAA TA		
*FSH-R*	F: AAA CTG GAA AAG ACA AAA CAC TG	60	228
	R: GGT CAA AAC CAA TGC CAT AG		
*PRL-R*	F: TCT GAA AGA TGC CAG GTA CAC	56	206
	R: TGC CCA GTC ATT TAT TGA CA		

### Data Analysis and Statistics Method

The test data were analyzed by SAS software (8.1), ANOVA by one-way and two-way ANOVA, and the differences were compared using the Duncan method. The test results were expressed as mean ± standard error, and *P* < 0.05 was used as the criterion to judge the difference.

## Results

### Reproductive Performance

There were no significant differences on day of first egg for Zi-goose reared with different color LED lighting. But day of first egg for Zi-goose provided with red LED lighting advanced 1 day more than those provided with blue and white LED lighting. Particularly, significant differences were found in the egg-laying peak duration, total egg number, total qualified egg number, and total average egg-laying rate for Zi-goose reared with different color LED lighting. The reproductive performance of Zi-goose treated with red LED lighting was higher than those treated with blue and white LED lighting (Table 3). There were no significant differences in these reproductive indexes between blue LED treatment and white LED treatment.

**Table 3:** Egg-laying peak duration, total egg number, total qualified egg number and total average egg-laying rate for Zi-geese under different color LED treatments.

**LED color**	**Day of first egg**	**Egg-laying** **peak duration** (week)*	**Total egg** **number**	**Total qualified** **egg number**	**Total average** **egg-laying rate**
Red	Mar.2	12a^*^	1,531a	1,373a	32.16a
White	Mar.3	9b	1,417b	1,236b	29.77b
Blue	Mar.3	9b	1,424b	1,250b	29.93b

* Here egg-laying peak duration represented week numbers when weekly average egg-laying rate was more than 30%.

*Different letters in the same column represented significant differences at 0.05 level.

**Figure 1:**
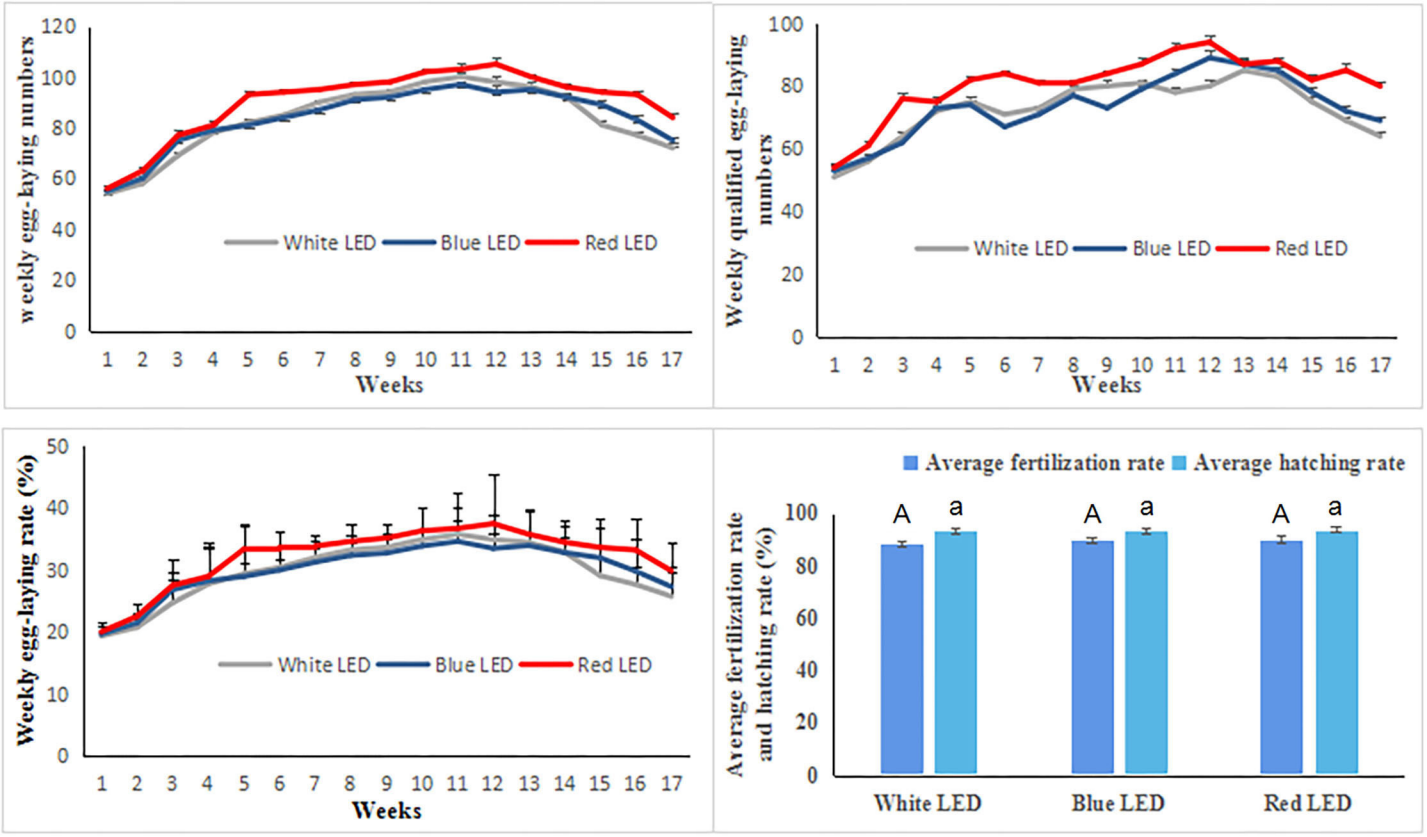
The weekly average egg-laying numbers, qualified egg-laying numbers, egg-laying rate, fertilization rates, and hatching rates of Zi-goose under different color LED treatments. The uppercase letter A indicates the difference significance of average fertilization rate while the lowercase letter a indicates for the difference significance of average hatching rate under different color LED illumination.

The weekly average egg-laying numbers, qualified egg-laying numbers, egg-laying rate, fertilization rates, and hatching rates were used to explore the reproductive performance of Zi-goose under different treatments. Under different color LED treatments, the reproductive performance indexes of Zi-goose are shown in Figure 1. The weekly average egg-laying numbers, qualified egg-laying numbers, and egg-laying rate of Zi-goose under red LED treatment were 90.06, 80.76, and 32.16%, respectively, which were significantly higher than those under white LED and blue LED treatments (*P* < 0.05). However, there were no significant differences in weekly average egg-laying numbers, qualified egg-laying numbers, and egg-laying rate of Zi-goose between blue LED treatment and white LED treatment (*P* > 0.05). The weekly average egg-laying numbers and egg-laying rate under blue LED treatment were subtly higher than those under white LED from week 15 to week 17, and the qualified egg-laying numbers under blue LED treatment were subtly higher than those under white LED light from week 11 to week 17. According to the data on the weekly average fertilization rate and hatching rate of Zi-goose (Figure 1), there were no significant differences in the weekly average fertilization rate and hatching rate of Zi-goose among different treatments (*P* > 0.05). These results indicated that red LED treatment was beneficial to improve the reproductive performance of Zi-goose, as evidenced by the increase in weekly average egg-laying numbers, qualified egg-laying numbers, and egg-laying rates.

### Effects of Different Color Illumination on Egg Quality of Zi-Goose

There was no significant difference in average egg weight, egg white ratio, egg yolk ratio, and eggshell ratio under different illumination (Table 4) (*P* > 0.05). However, different treatments had significant effects on yolk color and Haugh unit (*P* < 0.05). Also, there were no significant effects on egg shape index, eggshell strength, and eggshell thickness (*P* > 0.05). The yolk color of blue light treated birds was significantly darker than that of white light (*P* < 0.05), while the yolk color of red light treated birds were somewhere in between. The Haugh units of birds under blue light treatment were significantly lower than that of white light (*P* < 0.05), and the Haugh units of red light treated birds were still in the middle level (Table 5).

**Table 4:** Effects of different color light on egg weight and its composition of Zi-goose.

**Light type**	**Egg weight (g)**	**Ratio of egg white** **to egg weight**	**Ratio of yolk to** **egg weight**	**Ratio of eggshell to** **egg weight**
White light	119.85 ± 9.51^a^	0.53 ± 0.05^a^	0.33 ± 0.05^a^	0.14 ± 0.01^a^
Blue light	121.43 ± 8.14^a^	0.53 ± 0.03^a^	0.33 ± 0.03^a^	0.14 ± 0.01^a^
Red light	119.30 ± 9.81^a^	0.52 ± 0.03^a^	0.33 ± 0.03^a^	0.14 ± 0.01^a^

That there are different alphabets at the same columns indicate significant differences at 0.05 level.

**Table 5:** Effects of different color light on egg quality of Zi-goose.

**Light type**	**Egg shape index**	**Yolk color**	**Eggshell strength (N)**	**Eggshell** **thickness (mm)**	**Harge unit**
White light	1.44 ± 0.10^a^	6.44 ± 0.92^b^	80.23 ± 15.34^a^	0.60 ± 0.05^a^	52.51 ± 8.45^a^
Blue light	1.45 ± 0.06^a^	6.93 ± 1.08^a^	81.13 ± 15.10^a^	0.61 ± 0.06^a^	48.63 ± 9.48^b^
Red light	1.46 ± 0.06^a^	6.78 ± 0.98^ab^	80.70 ± 13.78^a^	0.60 ± 0.05^a^	50.84 ± 5.92^ab^

That there are different alphabets at the same columns indicate significant differences at 0.05 level.

### Effects of Different Color Illumination on Reproductive Hormone Levels in Zi-Goose

**Figure 2:**
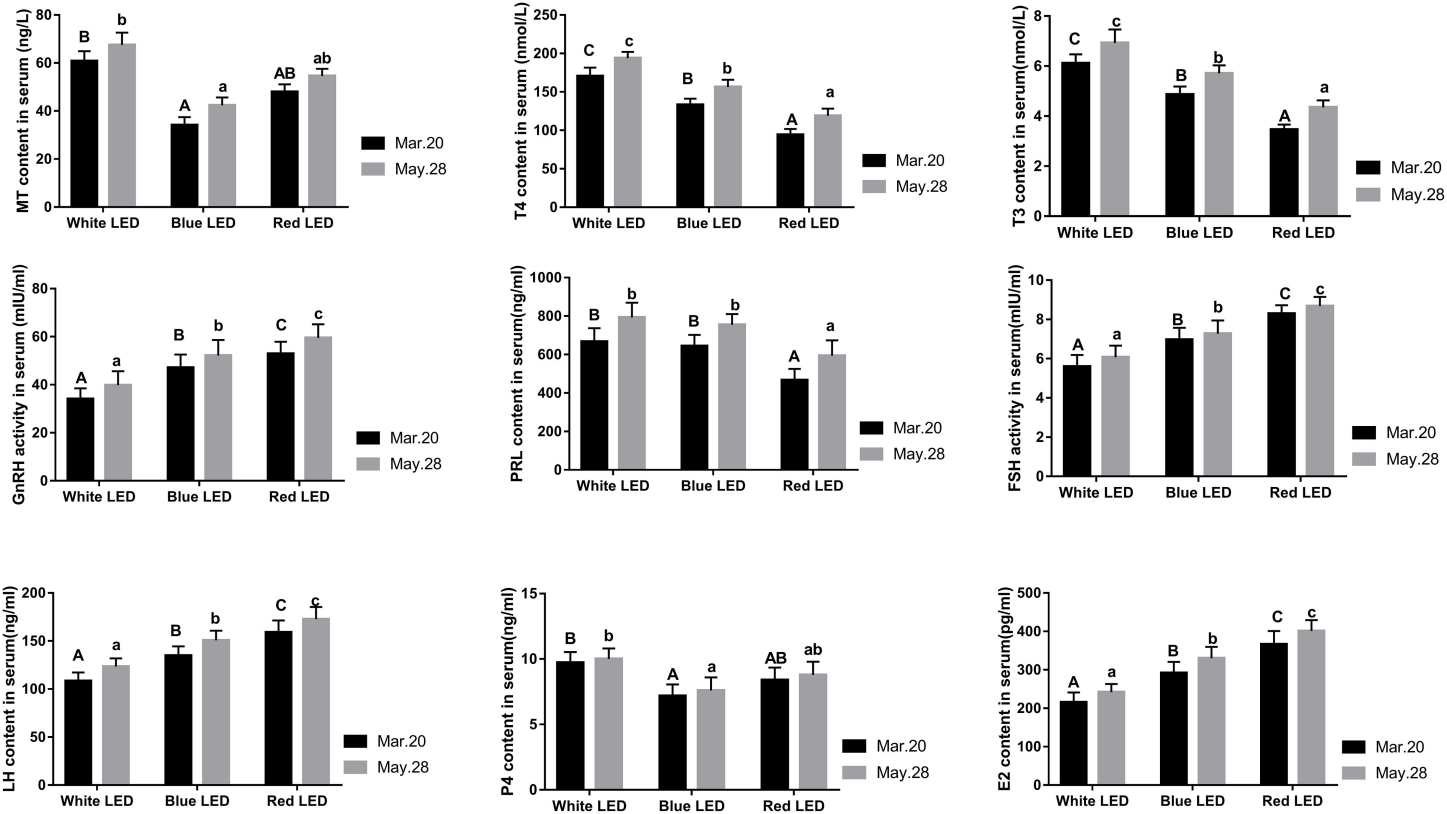
Effects of different color LED illumination on serum hormone levels of Zi-goose. The columns containing different letters in the figure represent significant differences at 0.05 level. Capital letters indicate significant differences among the average egg-laying number, and small letters indicate significant differences among the average eligible egg-laying number.

The changes in reproductive hormone levels in serum under different color LED treatments are shown in Figure 2. It is evident that the serum GnRH, FSH, LH, and E2 levels under red LED treatment were significantly higher than those under blue LED treatments (*P* < 0.05), while these serum hormone levels under blue LED treatment were significantly higher than those under white LED treatments (*P* < 0.05). However, the serum PRL, T3, and T4 levels under red LED treatment were significantly lower than those under blue LED treatment (*P* < 0.05), while these serum hormone levels under blue LED treatment were significantly lower than those under white LED treatments (*P* < 0.05). Although the serum MT and P4 levels under red LED treatment were slightly higher than those under blue LED treatment while lower than those under white LED treatment (*P* > 0.05), these hormone levels under blue LED treatment were significantly lower than those under white LED treatment (*P* < 0.05). Meanwhile, there was no significant difference in serum PRL level between white LED treatment and blue LED treatment (*P* > 0.05). It could be concluded that red LED illumination was more effective to improve the serum GnRH, FSH, LH, and E2 levels and decrease serum PRL, T3, and T4 levels of Zi-goose, which were the main reasons for improving its reproductive performances. Although blue LED lighting could also promote the synthesis and secretion of GnRH, FSH, LH, and E2, the synthesis and secretion of PRL, T3, T4, P4, and MT were significantly decreased (*P* < 0.05), which resulted in the nearly similar egg laying performance between blue LED and white LED lighting, but slightly improved in the later laying period.

### Transcriptome Sequencing Analysis

#### Screening of Differentially Expressed Unigene

**Figure 3:**
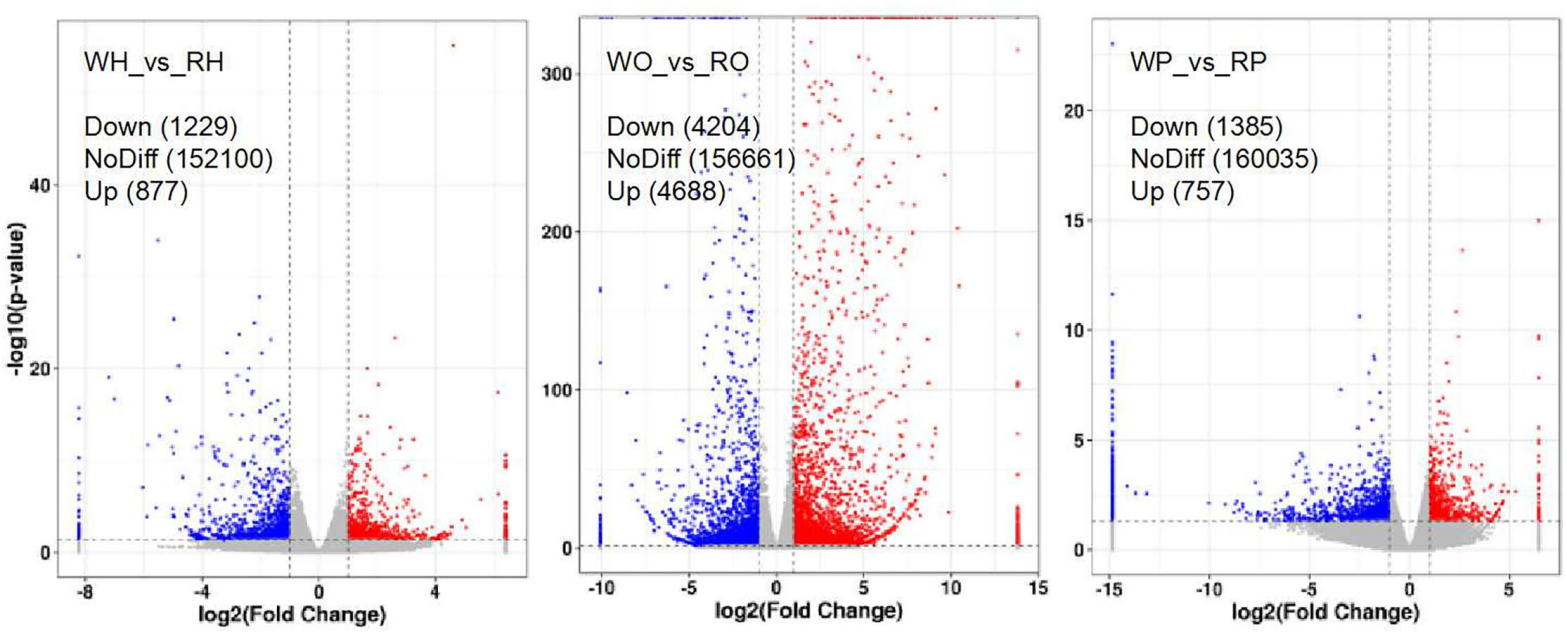
Volcanic map of differentially expressed genes. The abscissa is log_2_ (Fold Change) and the ordinate is –log_10_ (*P*-value). In the figure, the two vertical dashed lines are twice the threshold of expression difference. The horizontal dashed line is the threshold of *P* = 0.05. Red dots indicate up-regulated genes in this group, blue dots indicate down-regulated genes in this group, and gray dots indicate genes that are not significantly differentially expressed.

After the samples underwent RNA extraction, purification, library construction, and sequencing, the original sequencing data were formed, and then the unigenes were obtained through quality control and splicing. The functional annotation and classification of unigenes and screening of differentially expressed unigenes were carried out. The results of screening differentially expressed unigenes are shown in Figure 3. We noticed 2,106 unigenes (in the hypothalamus of Zi-goose) which showed differences in expression due to different color LED treatments. Of these, 1,229 unigenes were down-regulated and 877, up-regulated.

#### Enrichment Analysis of Differentially Expressed Genes by GO Analysis

**Figure 4:**
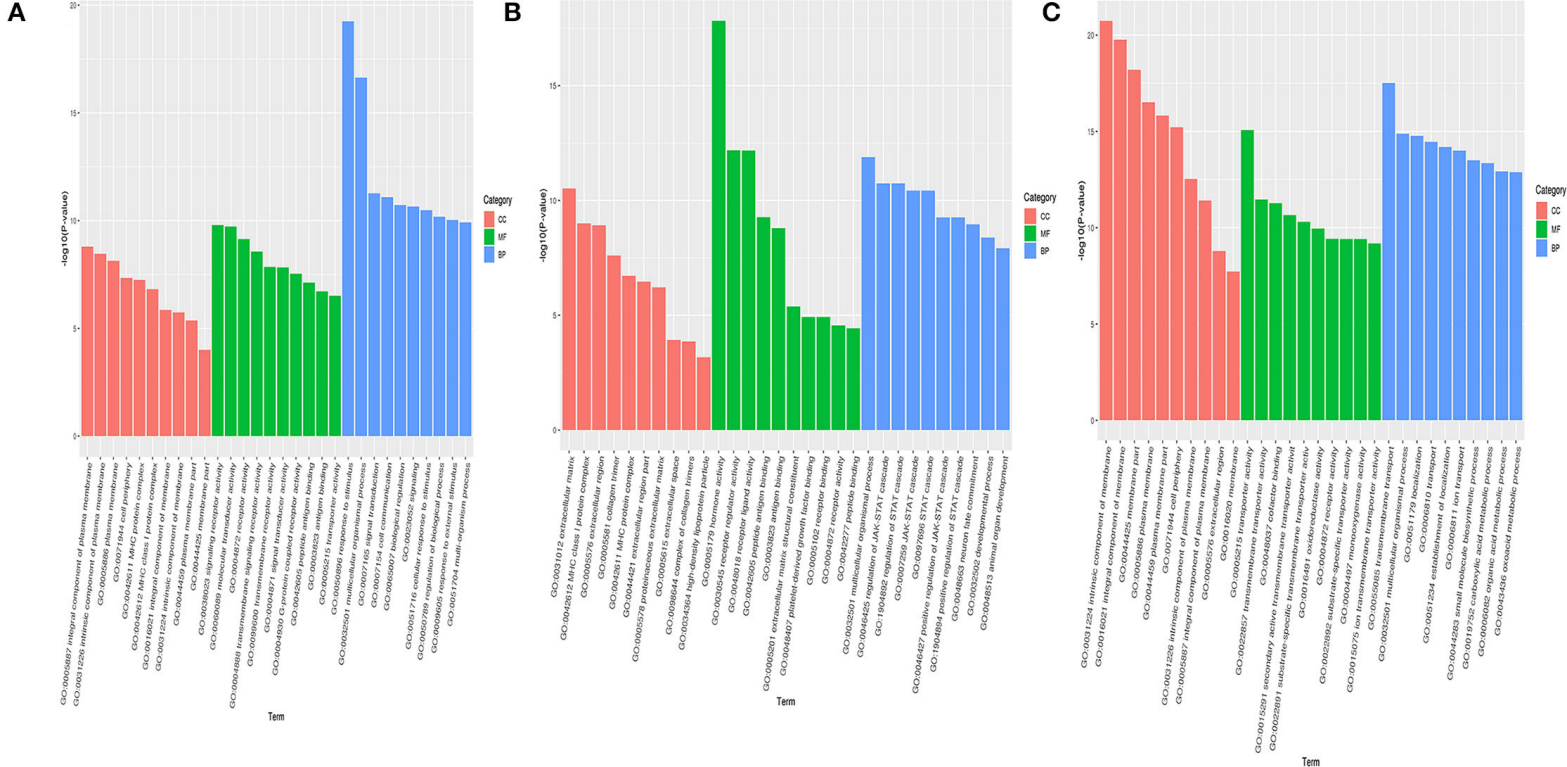
Hypothalamus, pituitary, ovary of GO enrichment analysis of differentially expressed genes in Zi-goose. Note: the abscissa is the term of Go level 2 grade, and the ordinate is the –log_10_ (*P*-value) enriched for each term. **(A–C)** represent the results of GO enrichment analysis of differentially expressed genes in hypothalamus, pituitary, ovary of Zi-goose.

The differentially expressed genes screened from the hypothalamus of Zi-goose were taken for the GO enrichment analysis. The top ten differentially expressed genes were associated with signal receptor activity, molecular sensor activity, receptor activity, transmembrane signal receptor activity, transmembrane receptor activity, signal receptor activity, G-protein coupling receptor activity, peptide antigen binding, antigen binding, and transporter activity, respectively. The top ten differentially expressed genes in BP classification were related to stimulus-response, multicellular biological process, signal transduction, cell communication, biological regulation, signal sending, cell response to stimulus, biological process regulation, external stimulus-response, and multi-organism process. The top ten differentially expressed genes with the most significant enrichment in cell component (CC) classification were classified as the overall component of plasma membrane, inherent component of plasma membrane, plasma membrane, cell periphery, MHC protein complex, level 1 MHC protein complex, overall component of membrane, inherent component of membrane, plasma membrane part, and membrane part respectively (Figure 4A). In Zi-goose pituitary screened for differentially expressed genes, the molecular functions (MF) classification enrichment of the most significant top ten differentially expressed genes and hormone activity respectively were: receptor activity, receptor ligands activity, peptide antigen, antigen, extracellular matrix structure composition, platelet-derived growth factor binding, peptide receptor, the receptor activity, and combining with related. The top ten differentially expressed genes were associated with multicellular biological processes, JAK-STAT cascade regulation, STAT cascade regulation, JAK-STAT cascade positive regulation, STAT cascade positive regulation, neuronal fate determination, developmental process, and animal organ development, respectively. The top ten differentially expressed genes with the most significant enrichment in CC classification belonged to extracellular matrix, level 1 MHC protein complex, extracellular region, MHC protein complex, extracellular region part, protein extracellular matrix, extracellular space, collagen trimer complex, and high-density lipoprotein particles (Figure 4B). Among the differentially expressed genes screened in Zi-goose nests, the MF classification enrichment of the most significant top ten differentially expressed genes was transporter activity, transmembrane transporter activity, auxiliary factor, secondary active transmembrane transporter activity, the substrate specificity of transmembrane transporter activity, REDOX enzyme activity, receptor activity, transporter substrate specificity, single oxygenase activity, and ion transmembrane transporter activity, The top ten differentially expressed genes were associated with transmembrane transport, multicellular biological process, localization, establishment of localization, ion transport, small molecule biosynthesis, carboxylic acid metabolism, organic acid metabolism, and oxyacid metabolism. The top ten differentially expressed genes with the most significant enrichment in CC classification belonged to the inherent component of membrane, whole component of membrane, membrane part, plasma membrane, plasma membrane part, cell periphery, inherent component of plasma membrane, whole component of plasma membrane, extracellular region, and the membrane category (Figure 4C).

#### Enrichment Analysis of Differentially Expressed Genes by KEGG Analysis

KEGG pathway enrichment analysis showed that the hypothalamus, ovary, and pituitary were involved in 279, 327, and 275 KEGG metabolic pathways in light response, respectively. Through further screening, the same eight metabolic pathways closely related to reproductive hormones were obtained in the hypothalamus, pituitary gland, and ovary (Tables 6–8).

**Table 6:** Metabolic pathways related to reproductive hormones in hypothalamus of Zi-geese.

**Pathway ID**	**Pathway name**	**Up DEGs**	**Down DEGs**	**DEGs no**.	***P*** **-value**	**FDR**
ko04912	GnRH signaling pathway	9	6	15	0.00	0.00
ko04913	Ovarian sterogenesis	0	1	1	0.88	1.00
ko04914	Oocyte maturation mediated by progesterone	1	0	1	0.99	1.00
ko04915	Estrogen signaling pathway	2	4	6	0.71	1.00
ko04916	Melanin formation	2	7	9	0.06	0.23
ko04917	Prolactin signaling pathway	1	3	4	0.59	0.93
ko04918	Thyroid hormone synthesis	2	5	7	0.05	0.21
ko04919	Thyroid hormone signaling pathway	1	8	9	0.28	0.65

**Table 7:** Metabolic pathways related to reproductive hormones in pituitary of Zi-geese.

**Pathway ID**	**Pathway name**	**Up DEGs**	**Down DEGs**	**DEGs No**.	***P*** **-value**	**FDR**
ko04912	GnRH signaling pathway	8	5	13	0.00	0.01
ko04913	Ovarian sterogenesis	2	3	5	0.04	0.21
ko04914	Oocyte maturation mediated by progesterone	0	3	3	0.81	1.00
ko04915	Estrogen signaling pathway	2	7	9	0.18	0.57
ko04916	Melanin formation	1	1	2	0.94	1.00
ko04917	Prolactin signaling pathway	1	18	19	0.00	0.00
ko04918	Thyroid hormone synthesis	0	3	3	0.58	1.00
ko04919	Thyroid hormone signaling pathway	0	3	3	0.95	1.00

**Table 8:** Metabolic pathways related to reproductive hormones in ovary of Zi-geese.

**Pathway ID**	**Pathway name**	**Up DEGs**	**Down DEGs**	**DEGs No**.	***P*** **-value**	**FDR**
ko04912	GnRH signaling pathway	10	12	22	0.52	0.94
ko04913	Ovarian sterogenesis	9	13	22	0.00	0.00
ko04914	Oocyte maturation mediated by progesterone	9	8	17	0.74	1.00
ko04915	Estrogen signaling pathway	15	20	35	0.10	0.33
ko04916	Melanin formation	14	12	26	0.09	0.33
ko04917	Prolactin signaling pathway	10	7	17	0.47	0.89
ko04918	Thyroid hormone synthesis	13	10	23	0.01	0.04
ko04919	Thyroid hormone signaling pathway	15	8	23	0.88	1.00

### Expression of Reproduction-Related Genes

**Figure 5:**
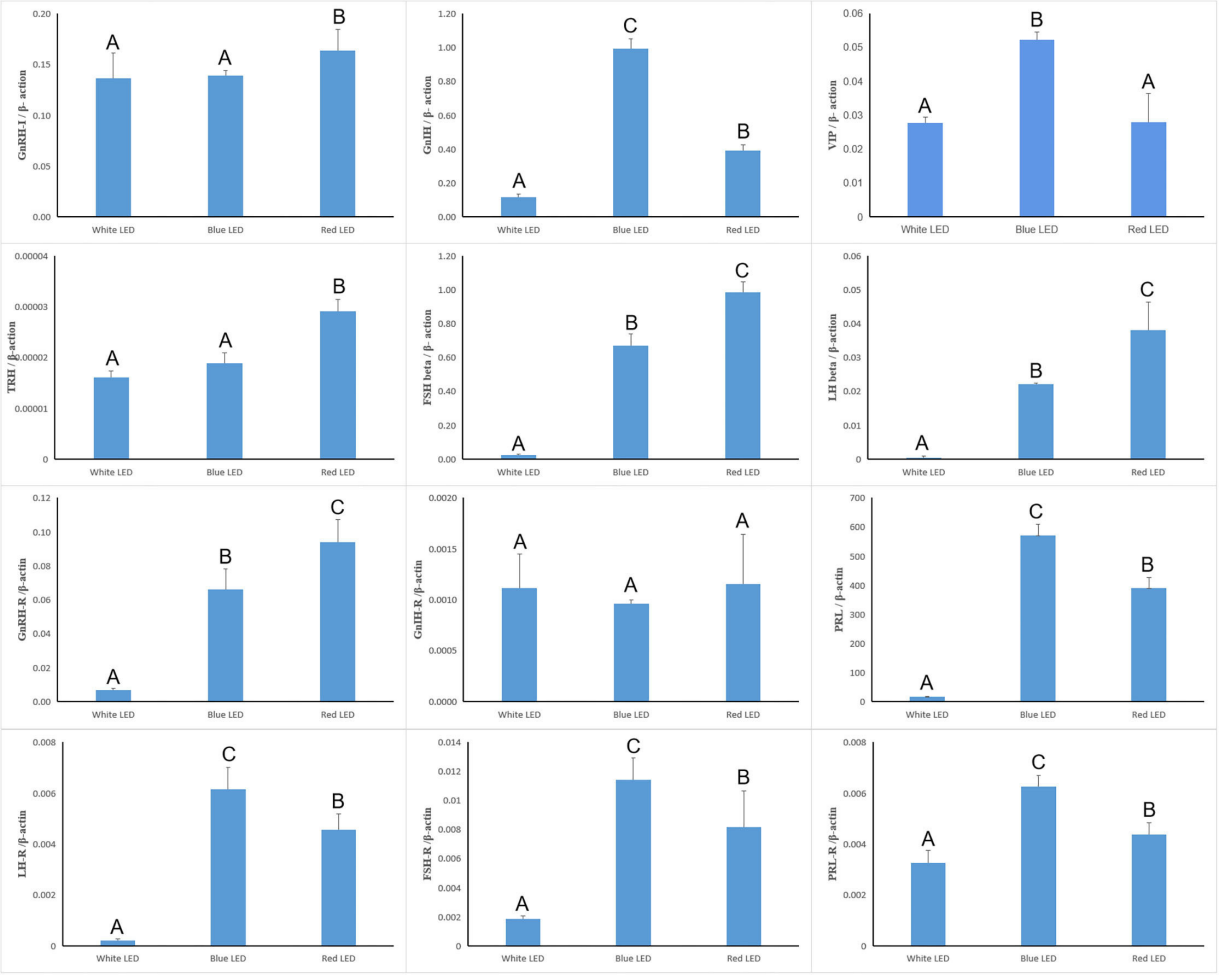
Real-time quantitative PCR of reproduction-related genes. The A–C letters indicate significant differences at 0.05 level.

The reproductive genes in the hypothalamus, pituitary, and ovary of Zi-goose were heavily affected by the light color (Figure 5). The expression of *GnRH-I, GnIH, TRH, LH* beta, *FSH* beta, *GnRH-R, PRL, LH-R, FSH-R*, and *PRL-R* mRNA of Zi-goose under red light treatment and the expression of *GnIH, VIP, LH* beta, *FSH* beta, *GnRH-R, PRL, LH-R, FSH-R*, and *PRL-R* mRNA of Zi-goose under blue light treatment were significantly up-regulated in comparison with those under white light treatment (*P* < 0.05). Compared with blue light treatment, the expression of *GnRH-I, TRH, LH* beta, *FSH* beta, and *GnRH-R* mRNA of goose treated with red LED lighting were significantly up-regulated while the expression of *GnIH, VIP, PRL, LH-R, FSH-R* and *PRL-R* of goose treated with red LED lighting were significantly down-regulated (*P* < 0.05). However, the light color had no obvious impact on the expression of *GnIH-R* mRNA of Zi-goose (*P* > 0.05).

## Discussion

Light wavelengths (light color) have an important impact on the reproductive performance of birds. When the light intensity and lighting time are accurately controlled, the effect of the light treatment on the reproductive performance of birds can be identified concisely (11, 12). In our experiment, the lighting time was set at 12 h/Day from 6:00 AM to 6:00 PM and the light intensity constantly remained at 10 Lux measured from one meter above the ground in the chamber, which proved to be the most effective for improving the reproductive performance of the goose in our preliminary experiment. The results indicated that the day of first egg for Zi-goose provided with red LED lighting advanced one day more than those provided with blue and white LED lighting. Despite no significant acceleration in sex maturity of Zi-goose, the red LED lighting showed a certain promotive role in the sex maturity of Zi-goose, which was in accordance with the results of Zhu et al. (13) and Hassan et al. (14). Our results confirmed that the red LED lighting could improve the egg-laying performances, showing longer egg-laying peak duration, higher total egg number, higher total qualified egg number, and higher total average egg-laying rate than those provided with blue LED and white LED lighting. Comparatively, the weekly average egg-laying numbers, qualified egg-laying numbers, and egg-laying rate of Zi-goose with red LED lighting were obviously higher than those treated with white LED and blue LED lighting. These results indicated that it was effective to improve egg-laying performances of Zi-goose by treating them with red LED lighting, which not only confirmed our hypothesis, but also corroborated the results of Gongruttananun et al. (15), Reddy et al. (16), and Zhu et al. (13). As for the favorable effects of red light on the reproductive performance of Zi-goose, we deemed that Zi-goose had a preferable perception and uptake on red light (long-wavelength Spectra), which are more efficient to penetrate and enter the brain and produce more intense stimulation to photoreceptors than blue light (short-wavelength Spectra), eventually enhancing reproductive function and increasing egg production in poultry.

It is known that photo simulation impacts the reproductive performances of birds by regulating the synthesis and secretion of reproductive hormones of the gonadal axis. Our results showed that red LED illumination was more effective to improve the serum GnRH (17), FSH (18), LH, and E2 (9) levels and decrease serum PRL, T3, and T4 levels of Zi-goose, which were the main reasons for improving its reproductive performances of Zi-goose (19, 20). Reddy et al. also found that when treated with red light, group laying performance was the highest, and the LH and GnRH concentration of laying hens was higher than in the blue light group (21). The results showed that blue light could stimulate the secretion of FSH and LH, improve the secretion in the Fallopian tube, and prolong the peak time of laying. Wang (22) points out that blue and green light promotes the secretion of the thyroid hormone and testosterone more than other monochromatic light. When Reddy et al. (23) treated leghorn chickens at the end of laying period (72–82 weeks) with red light, the concentrations of serum LH and E2 were significantly increased. Baxter et al. also found that egg-laying hens treated with red light for 6 weeks had significantly increased peripheral blood E2 levels at 20 weeks of age (9).

RNA sequencing can not only sort and classify gene expressions but also compare them (24). Previous studies have suggested that all cells of an individual of any species should contain the same DNA, but each organ, tissue, and even cell has a slightly different RNA expression, which allows different organs, tissues, and cells to perform different functions (25). Therefore, to explain these differences, we need to study RNA in these different organs, tissues, and cells, which requires RNA sequencing, or transcriptomics. In this study, to understand the impact of photo simulation on the reproductive performance of Zi-goose, transcriptomic research techniques were used to analyze the reproductive organ samples of Zi-goose during the peak laying period, so as to identify the possible genes and regulation mode of red light for 12 h to improve the reproductive performance of Zi-goose. Transcriptome research methods mainly include hybridization technology represented by cDNA chip and oligonucleotide chip, sequencing technology represented by SAGE, MPSS, and RNA-seq high throughput sequencing technology. Transcriptome research based on hybridization technology has disadvantages such as low sensitivity and difficult detection of microarray technology. While it is suitable for the detection of known sequences, it cannot capture new mRNA. SAGE and MPSS based on sequencing technology also have problems of long analysis time, relatively low throughput, and high cost, and are gradually getting replaced by RNA-seq based on high-throughput sequencing technology. RNA-seq research technology has the advantages of fast analysis, high throughput, low cost, and direct transcriptome analysis without understanding the genetic information of species, which is particularly important for the study of non-model organisms (26). In this study, we used Next-Generation Sequencing (NGS) for parametric transcriptome analysis based on the Illumina Sequencing platform. According to the raw data obtained, the base quality of the 5^′^ and 3^′^ ends of all Reads was low, and the base quality of the middle part was high. The base mass of most sequences was more than 20, indicating good sequencing quality. The average quality distribution of Reads also showed a high peak tip corresponding value and no trailing peak, indicating good overall sequencing quality, which not only proved that the sequencing technology we adopted was reasonable and effective but also provided the necessary basic guarantee for differential gene screening and functional analysis.

This study showed that red light for 12 h could significantly improve the reproductive performance of goose, which was manifested by the improvement of serum GnRH, FSH, LH, P4, E2, MT, T3, and T4 levels of female goose. Transcriptomic analysis showed that 2,106 unigenes were differentially expressed in the hypothalamus of birds under 12 h red light and 12 h white light, among which 1,229 unigenes were down-expressed and 877 were up-expressed. There were 8,892 unigenes differentially expressed in light color in Zi-goose egg nests, including 4,204 down-expressed and 4,688 up-expressed. There were 2,142 unigenes differentially expressed in the hypophysis of goose, including 1,385 down-expressed and 757 up-expressed. These differentially expressed genes involved 279, 275, and 327 KEGG metabolic pathways in the hypothalamus, pituitary, and ovary, respectively. Further screening showed the presence of GnRH signaling pathways in the Zi-goose hypothalamus, GnRH signaling pathways and PRL signaling pathways in the Zi-goose pituitary, and ovarian steroidogenesis and thyroid hormone synthesis pathways in the Zi-goose egg nest. These are closely related to the goose's reproductive hormones and genes and regulatory factors involved in these pathways are an important factor to improve the performance of breeding Zi goose.

Also, ten candidate genes related to reproductive performance were identified. Two candidate genes were from the hypothalamus (*VIP* and *GnRH-1*), five candidate genes were from the pituitary (*GnIH, PRL, GnRH-R, FSH* *β*, and *LH* *β*), and three candidate genes were from the ovary (*PRL-R, FSH-R*, and *LH-R*). The expression levels of *GnRH-1* in the hypothalamus, *GnRH-R, FSH* *β*, and *LH* *β* in the pituitary, FSH-R and LH-R in the ovary under red light for 12 h were higher than those under white light for 12 h. On the other hand, the expressions of hypothalamus *VIP*, pituitary PRL, and ovary PRL-R candidate genes were lower under red light 12 h than those under white light 12 h. These results showed that the expression of *GnRH-1, GnRH-R, FSH* *β*, *LH* *β*, *FSH-R*, and *LH-R* were up-regulated, and *VIP, PRL*, and *PRL-R* were down-regulated by red light 12 h. The secretion and activity of reproductive hormones were also regulated, which led to the improvement of reproductive performance. These results were consistent with the findings of Mobarkey et al. (27) and Reddy et al. (21).

## Data availability statement

The original contributions presented in the study are publicly available. This data can be found here: https://www.ncbi.nlm.nih.gov/sra/PRJNA902280.

## Author contributions

ML: research concept, methodology, data extraction, analysis, and writing the draft. CL: resource searching, verification, formal analysis, supervision, and manuscript reviewing and editing. XZ: resources, methodology, project administration, supervision, and manuscript reviewing and editing. GL, YZ, and SY: resource searching and manuscript reviewing and editing. ML and ZZ: methodology and manuscript reviewing and editing. All authors contributed to the article and approved the submitted version.

## Funding

This research study was supported by the research business expenses of the scientific research institutes of Heilongjiang Province (No. CZKYF2021B003?, National Modern Waterfowl Industry Technical System Special Project (No. CARS-42-24).

## Conflict of Interest

The authors declare that the research was conducted in the absence of any commercial or financial relationships that could be construed as a potential conflict of interest.

## Publisher's Note

All claims expressed in this article are solely those of the authors and do not necessarily represent those of their affiliated organizations, or those of the publisher, the editors and the reviewers. Any product that may be evaluated in this article, or claim that may be made by its manufacturer, is not guaranteed or endorsed by the publisher.
